# Quantification and targeting of elusive neurotoxic amyloid oligomers

**DOI:** 10.1016/j.xcrm.2022.100636

**Published:** 2022-05-17

**Authors:** Nemil Bhatt, Rakez Kayed

**Affiliations:** 1 Department of Neurology, Neuroscience and Cell Biology, University of Texas Medical Branch, Galveston, TX 77555, USA; 2 Mitchell Center for Neurodegenerative Diseases, University of Texas Medical Branch, Galveston, TX 77555, USA

## Abstract

A study by Kass et al.^1^ aims to understand the size distribution of amyloid β oligomers in postmortem brain tissues from individuals with Alzheimer’s disease (AD) and AD mouse models. Moreover, they show a dose-dependent oligomer elimination by the RD2 compound.

## Teaser

A study by Kass et al.^1^ aims to understand the size distribution of amyloid β oligomers in postmortem brain tissue from individuals with Alzheimer’s disease (AD) and AD mouse models. Moreover, they show a dose-dependent oligomer elimination by the RD2 compound.

## Main text

An estimated 6.5 million people are living with Alzheimer’s disease (AD) in the United States, and the number of affected individuals is expected to double by 2050.^2^ Despite the rising prevalence, a cure for AD is still not available because of its complex nature.^2^ Although the mechanism of disease pathology is widely debated; AD is pathologically distinguished by the accumulation of amyloid β (Aβ) in senile plaques and tau into hyperphosphorylated neurofibrillary tangles.^3^ In addition, aggregates of other amyloidogenic proteins, such as TAR DNA-binding protein-43 (TDP-43) and α-synuclein, have also been found in AD, complicating its etiological landscape.^3^, ^4^, ^5^

Previously, it was believed that large, insoluble aggregates of amyloidogenic proteins caused neuronal dysfunction.^3^^,^^5^ However, accumulating evidence over the last 20 years suggests that it is the soluble multimers of these proteins, termed “oligomers,” that initiate the neuronal dysfunction in neurodegenerative diseases.^3^, ^4^, ^5^, ^6^ Aβ oligomers are largely present in diseased individuals^3^^,^^7^ and are reported to induce toxicity by increasing membrane permeability and promoting disruption in ion homeostasis.^3^^,^^6^^,^^7^ This suggests that soluble oligomers may be more neurotoxic compared to their large, insoluble counterparts.^3^^,^^4^

Oligomers of amyloid proteins can form a sequence-independent conformation, suggesting a similar mechanism of aggregation and toxicity.^6^ Moreover, the proteins listed above can co-aggregate in individuals with AD.^5^ Despite different proteins showing varying aggregation propensities, the two major groups of assemblies that have received much attention are soluble oligomers and large, insoluble aggregates.^3^, ^4^, ^5^^,^^7^ In the case of soluble oligomers of Aβ, dimers, trimers, tetramers, pentamers, decamers, and Aβ∗56, are all reported in affected individuals.^3^^,^^7^ In line with this hypothesis, an exciting study by Kass et al.^1^ analyzes the concentration and size distribution of Aβ oligomers in different transgenic mouse models and human AD brain samples using surface-based fluorescence intensity distribution analysis (sFIDA) in conjunction with density gradient centrifugation.^1^ The advantage of this approach lies in its ability to keep all the aggregates in a native state. It is worthy to note that although the oligomer concentration varies among individuals with AD, the oligomer size distribution remains similar among the samples.^1^ Additionally, the size distribution of these oligomers in mouse models is analogous to human AD samples.^1^ Similar Aβ oligomer size distribution has also been reported previously with other methodologies, further endorsing the work done by Kass et al.^3^ In addition, the size of the oligomer plays a crucial role in toxicity. Based on the published literature, there is an inverse relationship between the size of Aβ oligomers, their hydrophobic nature, and the induced toxicity.^3^ As the oligomers become larger in size, the surface hydrophobicity of the aggregates decreases and so does the potential toxicity exerted by these aggregates on the cells.^3^ A similar trend has been reported with soluble tau oligomers as well.^4^^,^^5^^,^^8^^,^^9^ Further research is ongoing to understand the structure and conformation of oligomers of different amyloidogenic proteins with varying sizes and their role in neurotoxicity. Thus, Kass et al. present an invaluable tool by combining sFIDA technique with particle-size-dependent fractionation to understand and characterize the nature of Aβ oligomers in human AD brain tissues. This may also be translatable to other disease-relevant oligomers, such as tau and α-synuclein oligomers.^1^

Fluorescent dyes provide an invaluable tool for monitoring the characteristic hydrophobicity and kinetics of amyloid aggregation. Thioflavin-T (Th T) is an example of such a dye, which fluoresces when bound to β-sheet-rich fibrillar amyloid structures and is routinely used to monitor fibril formation. Several groups have reported, as reviewed by Sengupta et al., that Th T-positive amyloid β fibrils are inert and non-toxic.^3^^,^^8^ 4,4′-Dianilino-1,1′-Binaphthyl-5,5′-Disulfonic Acid (bis-ANS) dye is another dye that serves as a probe for measuring surface hydrophobicity of amyloids and is routinely used to characterize oligomers. For example, Aβ and tau aggregates, which are bis-ANS positive, are more cytotoxic compared to their Th T-positive aggregated forms.^3^^,^^8^ Continuing this line of research and the work done by Kass et al. is essential to understand the correlation between the biophysical and biochemical characteristics of amyloidogenic proteins and their propagation.^1^

Recent research also focuses on the spreading of amyloid protein aggregates, including Aβ, tau, synuclein, and TDP-43, believed to occur in a prion-like manner, which may explain the spread of AD pathology to different regions of the brain.^4^^,^^5^ These studies suggest the spreading and propagation of amyloid aggregates as a common component in disease pathologies.

In line with the evidence mentioned above, targeting oligomeric forms of amyloidogenic proteins may prove useful in halting disease spreading in AD and other neurodegenerative diseases.^9^ Targeting oligomers through immunotherapy is in preclinical stages. However, to date, there has been no success of immunotherapy in clinical trials, and it needs to be investigated more thoroughly to establish its safety and efficacy.^9^

Small molecules, for example, curcumin derivatives, represent one such therapeutic avenue and can be used to target tau oligomers. Curcumin derivatives can be used to reduce cytotoxicity by changing the conformation of tau aggregates.^8^ The curcumin derivatives bind to tau oligomers and promote them into large, non-toxic insoluble aggregates by changing their biophysical and biochemical characteristics.^8^ Following a similar underlying theme, a different approach is presented in the study by Kass et al. The approach outlined in the study is to use RD2 to dissemble Aβ oligomers into monomers.^1^ Both approaches mentioned above target soluble oligomers and aim to generate non-toxic counterparts, albeit via different mechanisms. Although more in-depth studies are still needed to provide the usefulness of RD2 as a therapeutic, Kass et al. show the dose- and time-dependent clearance of Aβ oligomers via their compound in murine and human AD brain homogenates. They do this with the aim of understanding Aβ oligomers and their interactions with RD2 so that it may provide translational value.^1^

The reports mentioned above and the study presented by Kass et al. cumulatively emphasize the importance of oligomer size on disease pathology and provide potential strategies for disease intervention.
